# Monthly Botanical Report

**Published:** 1811-01

**Authors:** 


### MONTHLY BOTANICAL REPORT.

Having for some Months past made no mention of English Botany, we shall now resume our usual account of such phœnogamic plants as occur from the 1st of July to the end of the year; of the cryptogamic plants, except those of the order of filices, we shall not take any notice.

Galium *verrucosum*, the Valantia *Aparine* of Linnæus. Dr. Smith has very properly swerved from his great masters in this instance: indeed the small importance of some of the flowers being defective in part of the sexual organs, is now much better understood than formerly. The true Valantias are distinguished by much more important characters in the seed. The figure of this plant as given by Mr. Sowerby is strikingly different from that of Vaillant, in the greater length and straightness of the peduncles; found by Mr. G. Don in corn-fields in the carse of Gowrie.

Juncus *gracilis*, supposed to be a non-descript species, also found by Mr. Don, among the mountains of Angus-shire, but very rarely It approaches to J. *bufonius*.

Caltha *radicans*; first described by T. F. Forster, esq. in the eighth volume of the Transactions of the Linnæan Society.

Pinguicula *grandiflora*, of Decandolle and Lamarck; sent from Ireland by the Rev. Mr. Hincks, Secretary to the Cork Institution; found plentifully in the western parts of the county of Cork, by Mr. Drummond.

Carex *pallescens*; common in most groves and pastures.

Salex *tenuifolia*; native of Westmoreland and Scotland, drawn from a specimen in the garden of Mr. T. F. Forster, at Clapton. The name is derived from the thinness of the substance of the leaf.

Brassica *rapa*, the common turnip. A valuable observation of Mr. J. A. Knight's, the celebrated vegetable physiologist, is inserted, proving that the Swedish turnip is a variety of this, and not of the cabbage, as has been supposed.

Sagina *maritima*; a minute plant, much resembling Sagina *apetala*, found on the sea-coast of Scotland and Ireland, and on the summit of Ben Nevis.

Rosa *hibernica*. Some patrons of Botany at Dublin offered a premium of fifty pounds for the discovery of a new Irish plant, which reward was claimed by J. Templeton, esq. in consequence of his discovery of this supposed non-descript species. Its character is "fruit nearly globose, (red) smooth as well as the flower-stalks. Prickles of the stem slightly hooked. Leaflets elliptical, smooth, with hairy ribs."

Fragaria

Fragaria *elatior;* the hautboy strawberry; found in a wood on the west side of Tring in Hertfordshire, and in Charlton Forest, Sussex. This species bearing male and female flowers on different roots is very apt to be unproductive even in a cultivated state. It should be the business of gardeners to take care that some barren or male plants are intermixed with the fruit-bearing ones, which would probably insure a plentiful crop.

Betula *alba;* the birch. Every admirer of picturesque beauty is acquainted with the elegance, as every schoolboy is with the disciplinarian virtues of this beautiful and useful tree.

Aspidium *irriguum;* supposed to be a new species of fern, discovered by Mr. J. F. Foster, about the margins of clear springs, near Tunbridge Wells. The drawing was taken from a garden specimen.

Galium *witheringii;* mistaken by Withering for the G. *montanum* of Linnæus.

Cistus *surrejanus.* This species is become a very dubious one, no wild specimen having been found since the time of Dillenius; the drawing was of course necessarily taken from a garden specimen.

Cistus *tomentosus;* of Scopoli. Dr Smith has received this from different botanists, gathered in Scotland, and discovers it to be the same as Scopoli's plant from a comparison of it with a specimen from that excellent botanist himself. Judging from the figures of the above two plants, they appear to us to differ only in the form of the petals and the nature of the pubescence on the under surface of the leaves; the difference of the former apparently arise from their being defective in *surrejanus,* and the latter, perhaps, solely from cultivation.

Scrophularia *Scorodonia,* a rare native of Jersey, and found also by Mr. E. Llwyd, about St Ives, in Cornwall; not yet observed in any other part of Great Britain; drawn from a garden specimen.

Hieracium *molle,* found by Mr. Dickson in woods in the south of Scotland. It agrees with authentic specimens from Jacquin in the Linnæan Herbarium.

Senecia *saracenicus;* one of the rarest of British plants; found in Yorkshire, Lancashire, and Westmorland.

Amaranthus *Blitum;* found in Battersea-field and elsewhere in the neighbourhood of London, on dunghills. Dr. Smith remarks, that it resembles Atriplex in habit more nearly than such of it's more specious congeners as decorate our gardens.

Avena *fatua;* a pernicious weed, especially infesting barley.

Frankenia *pulverulenta;* a very doubtful British species, drawn from a garden specimen, said to have been found on the coast of Sussex in the time of Dillinius, and Hudson professed to have gathered it himself between Bogner and Brighthelmstone.

Atriplex *erecta;* this species, at first rightly defined by Hudson, but afterwards improperly joined by him with *patula,* has not been of late found by any botanist, and hence has necessarily been figured from a dried specimen in Mr. Rose's herbarium, named under the inspection of Mr. Hudson.

Polypodium

Polypodium *Phegopteris*; a beautiful, delicate fern, growing in rather moist places, on mountains in the south of Scotland and north of England.

Of the Botanist's Repository we have received only one number since our last account of this work. The contents are,

Ipomæa *pendula*; native of New Holland, about Port Jackson as well as the tropical parts. It appears to be a very beautiful species, corollas large, flesh-coloured. The drawing was taken at the Comptesse de Vandes collection at Bayswater.

Fumaria *nobilis*; communicated by Mr. Donn, from the Botanic gaden at Cambridge, at present one of the first collections in Europe.

Globba *purpurea*. The mantisia *saltatoria* of the Botanical Magazine; drawn at Sir Abraham Hume's, from whence Mr. Leo's collection was supplied with it.

Euphorbia *epythymoides*; communicated by Mr. Donn, from the Cambridge garden. Native of Austria. The herbaceous euphorbiæ look so differently at different periods of their growth, that it is often difficult to determine the species, but from the very entire edges of involucre and the roundness of the leaves, we are inclined to doubt if this be the same as has been described and figured by Jacquin in the Flora Austriaea.

Euphorbia *meloformis*. A much better figure of this plant, though uncoloured, is to be seen in the Annales du Museum d'Histoire Naturelle, and copied from thence in the first volume of Annals of Botany, pl. 2. It is a diæcious plant, and we believe the male only has been as yet seen in this country.

The Botanical Magazine for last month contains

Aloë *rigida* of Decandolle, the *expansa* of Haworth.

Aloë *pentagona* of Haworth.

Anthericum *longiscapum* of Jacquin; from Mr. Haworth's collection. This, according to Mr. Ker, is the *asphodeloides* of the late edition of the Hortus Kewensis, as is proved by the specimen preserved in the Banksian Herbarium. It is not, however, the *asphodeloides* of Linnæus, Miller, &c.

Tradescantia *erecta*, an annual plant; native of Mexico.

Fothergilla *alnifolia*, var. *obtusa*, and var. *major*. Dr. Sims describes another variety under the name of serotina. This genus was named in honor of Dr. John Fothergill, by the late Dr. Garden of Charlestown, South Carolina. For an interesting life of the last mentioned author, by Dr. Smith, See Dr. Rees's Cyclopædia, article, Garden.

Arctolis *glutinosa*, a new species, as appears, though Dr. Sims is not certain with respect to the genus to which it ought to be referred; drawn at Lee and Kennedy's Museum, Hammersmith.

Phlox *Carolina*; an old inhabitant of our gardens, but probably for some time lost, and now recovered by Mr. Fraser, of Sloane-square. The smooth leaves and rough stem united seem to be sufficient to distinguish this from every other known species.

